# Does Executive Function Training Impact on Communication? A Randomized Controlled tDCS Study on Post-Stroke Aphasia

**DOI:** 10.3390/brainsci12091265

**Published:** 2022-09-19

**Authors:** Francesca Pisano, Alessio Manfredini, Andrea Castellano, Carlo Caltagirone, Paola Marangolo

**Affiliations:** 1 Department of Humanities Studies, University Federico II, 80133 Naples, Italy; 2 Department of Psychology, University La Sapienza, 00185 Rome, Italy; 3 IRCCS Santa Lucia Foundation, 00179 Rome, Italy

**Keywords:** tDCS, neuromodulation, functional communication, aphasia, executive functions

## Abstract

New approaches in aphasia rehabilitation have recently identified the crucial role of executive functions (EFs) in language recovery, especially for people with severe aphasia (PWSA). Indeed, EFs include high-order cognitive abilities such as planning and problem solving, which enable humans to adapt to novel situations and are essential for everyday functional communication. In a randomized double-blind crossover design, twenty chronic Italian PWSA underwent ten days of transcranial direct current stimulation (tDCS) (20 min, 2 mA) over the right dorsolateral prefrontal cortex (DLPFC). Two conditions were considered, i.e., anodal and sham, while performing four types of cognitive training (alertness, selective attention, visuo-spatial working memory, and planning), all of which were related to executive functions. After anodal tDCS, a greater improvement in selective attention, visuospatial working memory and planning abilities was found compared to the sham condition; this improvement persisted one month after the intervention. Importantly, a significant improvement was also observed in functional communication, as measured through the Communication Activities of Daily Living Scale, in noun and verb naming, in auditory and written language comprehension tasks and in executive function abilities. This evidence emphasizes, for the first time, that tDCS over the right DLPFC combined with executive training enhances functional communication in severe aphasia.

## 1. Introduction

The traditional model of language organization, often referred to as the Broca–Wernicke–Lichtheim–Geschwind model [1,2], focuses almost exclusively on the involvement of the inferior frontal and posterior temporal regions for expressive and receptive language functions, respectively, and the connection between these sites, named the arcuate fasciculus. However, in recent years, behavioral and neuroimaging results have shown that the network subserving the language function is much more widely distributed across the brain than previously thought [3,4,5]. Indeed, most contemporary models propose a much more complex architecture encompassing regions which might also include bilateral cortical networks as well as subcortical circuits [6,7,8,9,10]. In line with this view, a growing body of evidence has led to the concept of “neuronal multifunctionality”, in which these complex neuronal circuits subserve both linguistic and non-linguistic information, creating dynamic cognition-language interactions in the brain [11,12]. Based on this perspective, new approaches in aphasia rehabilitation have emphasized that non-linguistic functions may also subserve language recovery. Accordingly, several works have already shown that multiple cognitive domains, including attention [13,14,15,16,17], memory [18,19] and executive functions, may improve communication and language performance in aphasia [20,21,22,23]. Since in persons with severe aphasia (PWSA), language does not always adequately meet the communicative needs of the individual [24], to communicate successfully, it is necessary to enhance skills and strategies that allow PWSA to bypass their limitations in everyday activities. The impact of inadequate strategic competence in everyday life in PWSA clearly points to the importance of training executive functions for successful functional communication in aphasia [25,26]. Indeed, executive functions (EFs) include high-order cognitive abilities such as cognitive flexibility, planning and problem solving which enable humans to achieve goals, to adapt themselves to novel everyday life situations, and to manage social interactions [27,28,29]. To date, several definitions, and theoretical models for EFs processing have been proposed which were specifically designed to achieve different research purposes ([30]). With regard to the relationship between executive functions and functional communication, clinically oriented models are the most suitable choice. Indeed, clinical models, which originate from clinical neuropsychology, tend to be comprehensive, including multiple sub-components of EF which correspond to the deficits observed in brain-damaged populations. Examples of such models include those by Lezak, Howieson, Bigler, and Tranel [31], Stuss [32], and Suchy [33]. In particular, Suchy and colleagues [30] have proposed a model which describes the relationship between executive functions and functional communication incorporating five inter-related executive domains: (1) planning and problem-solving skills based on working memory and mental flexibility; (2) initiation and continuation of the behaviors necessary to implement a given action; (3) response selection, i.e., the ability to choose an appropriate action among several competitors, based on the processes of inhibition and updating; (4) multitasking, i.e., monitoring and coordinating multiple goals in a prospective view; (5) social cognition, i.e., understanding socially relevant verbal communication and paralinguistic messages. Thus, the authors consider the importance of executive functions to accomplish several communication skills [30]. As an example, while holding a conversation, the speaker should at the same time store the interlocutor’s information, plan the responses to be given, and sometimes inhibit inappropriate ones, all tasks implemented by EF processing.

Consistent with this hypothesis, several lines of evidence have already emphasized how executive functions could provide support to conversational skills when spared but they might also interfere with functional communication when impaired [23]. Indeed, different reports to date have clearly shown that PWA may also present with executive deficits, which negatively affect language treatment outcomes [20,34,35] and communication skills [25,26]. 

In recent years, new technological advances have shown the effectiveness of transcranial direct current stimulation (tDCS), a non-invasive brain stimulation technology, in the recovery of several cognitive abilities, including language recovery [36,37]. Through tDCS a weak current at low intensity (1–2 mA) is delivered on the scalp by means of two electrodes: the anode and the cathode [38]. Depending on the polarity of the current, there is a general agreement that anodal stimulation causes a depolarization of the neural membrane resulting in excitability of the target area, whereas cathodal stimulation might induce hyperpolarization with an inhibition of the activity of the stimulated region [39]. Typically, these two conditions (anodal, cathodal) are compared with a sham condition (placebo condition), in which the stimulator is switched off after 30 s without the subject’s awareness [36,40]. 

It is well known that the dorsolateral prefrontal cortex (DLPFC) is involved in higher-level cognition and, in particular, in domain general executive functional control, such as in selective attention, working memory and planning tasks ([41,42,43,44]; for a review, see [45]). With regard to selective attention, a couple of works have indicated an increase in response inhibition ability after anodal tDCS over the left DLPFC [46], while others have more recently emphasized the effectiveness of anodal tDCS over the DLPFC ([47,48,49,50]. In the context of WM, while some studies have successfully targeted the left DLPFC in verbal working memory [44,51,52], others have addressed the role of its right homologous [53,54] in spatial working memory tasks [54,55,56]. As regard as planning abilities, Harty and colleagues [57] have investigated the effects of both anodal and cathodal stimulation over the right and left DLPFC in a group of 106 healthy elderlies who presented a low error awareness measured through the Error Awareness Task [58]. Results showed that anodal tDCS over the right DLPFC exerted the greater effect increasing error awareness compared to the other conditions. In a sample of fifty-five healthy subjects, Heinze and colleagues [59] investigated the effects of bilateral tDCS over the DLPFC combined with an eye-tracking while performing a planning task by using the Tower of London test. Results showed a reduction in initial thinking time following left cathodal/right anodal DLPFC stimulation in parallel with a shorter duration of the last gaze before task’s solution. Based on these findings, the authors concluded that anodal stimulation over the right DLPFC is associated with a reduction in the time spent in evaluation processes during planning tasks. 

Although the DLPFC has never been considered as specifically related to language tasks, its role for implementing functional connectivity between the language network and other cognitive domains has been widely recognized (for a review, see [45]). Thus, several studies have stimulated the left DLPFC to improve error detection in sentences [60] or to reduce interference in picture naming tasks [61]. Very recently, Pestalozzi and collaborators [62] have investigated whether strengthening executive control through anodal tDCS over the left DLPFC would facilitate lexical retrieval in a group of fourteen PWA. Results showed an increase in verbal fluency and in naming speed of high frequency words after anodal condition with respect to sham. 

As far as we know, to date, none of the reported studies have investigated whether tDCS over the DLPFC combined with executive function training improves functional communication in PWSA. 

As previously stated, several lines of evidence have already highlighted the role of executive functions in maintaining an adequate level of communication skills in aphasic patients, particularly, in severe cases [20,25,26,34,35]. 

Thus, in the present study, we investigated whether anodal tDCS over the right DLPFC combined with different executive function training would enhance the ability to communicate in everyday life in twenty PWSA. The choice to stimulate the right DLPFC is pertinent with all the most recent literature indicating this region as crucial for the performance of executive tasks.

## 2. Materials and Methods

### 2.1. Study Design

A randomized double-blinded cross-over design was conducted from January 2020 to June 2022 at the Behavioral Neurological Laboratory of the IRCCS Santa Lucia in Rome, Italy. Thirty chronic persons with post-stroke aphasia were examined through a detailed neuropsychological assessment. Ten were excluded for the following reasons: failure to meet the inclusion criteria, difficulty in transporting means and personal reasons. Thus, a final sample size of twenty patients was recruited (see Figure S1).

G*Power 3.1 [63] was used to calculate the sample size with α = 0.05, a power = 90%, two measurements (anodal vs sham), and effect size f = 0.4. The analysis indicated that a total sample size of N ≥ 19 was necessary to detect a significant effect in our study.

All twenty patients received both interventions (AB → anodal-sham and BA → sham-anodal). The order of conditions was randomized across subjects. Half of the participants (n = 10) started with condition A (anodal tDCS) followed by condition B (sham tDCS), while the other half began (n = 10) with condition B (sham tDCS) followed by condition A (anodal tDCS). The allocation sequences were generated by a technician of the laboratory. To avoid carryover effects, a washout period of four weeks was established between condition A and B (and vice versa). As this was a double-blinded study, both the examiner and the patient were blinded regarding the stimulation condition and the stimulator was turned on/off by a third person, who assigned participants to the AB or BA intervention.

### 2.2. Participants

Twenty left-brain-damaged participants (ten men and ten women, mean age: 61.04; SD 7.02) with severe chronic aphasia were included in the study. Inclusion criteria were native Italian proficiency, a single left ischemic stroke at least 6 months prior to the investigation, pre-morbid right handedness (based on the “Edinburgh Handedness Questionnaire”; [64]) and no acute or chronic neurological symptoms needing medication. Subjects over 75 years of age and those with seizures, implanted electronic devices (e.g., pacemaker) and previous brain damage were excluded. None of the participants were taking any kind of medication and none of them has received structured language therapy for at least 6 months before the time of inclusion in the study in order to prevent confounding therapy effects (see Table 1). 

### 2.3. Ethics Statement

The data analyzed in the current study were collected in accordance with the Declaration of Helsinki and the Institutional review board of the IRCCS Fondazione Santa Lucia, Rome, Italy. Before participation, all patients signed informed consent forms.

### 2.4. Clinical Data

All patients were diagnosed with severe non-fluent aphasia. Subjects were not able to spontaneously speak, as their verbal output was totally absent. The aphasic disorders were assessed using standardized language testing (Esame del Linguaggio II (EDL), [65] and the Token Test [66]. The EDL test included different tasks among which oral and written noun and verb-naming (n = 20 for noun naming, i.e., topo (mouse); n = 10 for verb naming, i.e., *leggere* (to read)), words repetition, reading and writing under dictation (words, n = 20, i.e., tavolo (table)), nonwords repetition, reading and writing under dictation (n = 20, i.e., *bo, fime, tarino*). Although some residual repetition and reading abilities were still present, all subjects were severely affected in all tasks including the auditory comprehension test (Token test cut-off 29/36) (see Table 1). Oral (n = 60, i.e., *la moto ha superato la macchina* (the motorbike passed the car) and written comprehension (n = 45, i.e., *le bambine sono applaudite dal bambino* (the girls are applauded by the boy) were further assessed through two BADA subtests (Batteria per l’analisi dei deficit afasici, [67]) which indicated a severe impairment in both tasks (see Table 2). To assess functional communication, we used the Italian version of CADL-2 [68], an ecological evaluation tool which consists of 50 items in the form of role-playing activities revolving around fictitious environments (e.g., going to the doctor, shopping at the grocery, making a phone call, asking for directions, driving a car), depicted by questions and pictures. All PWA obtained a low percentage score, indicating a poor level of functional communication (0−23 low percentage scores, 24–77 average percentage score, 78–100 high percentage score). To investigate executive functions processing, all participants were also administered the attention visual search test [69] (cut-off score 30), the spatial short-term memory Corsi test ([70] (cut-off score 3.08), the non-verbal Smirni subtest which measures the ability to recognize previously presented faces and buildings ([71] (cut-off percentage score >10) and, for planning abilities, the Tower of London test (TOL, [72] (cut-off percentage score 80). In the visual search task, all patients performed close to the cut-off score, while in the Corsi test their performance was not impaired. In the Smirni test and in the TOL test, all patients were below the cut-off percentage score (see Table 2).

### 2.5. Materials

Cogniplus software (Schuhfried, https://www.schuhfried.com/cogniplus/ (accessed on 15 January 2020), Mödling, Austria, Europe), a cognitive battery for training different cognitive abilities embedded in lifelike scenarios, was used. Four cognitive tasks were selected: alertness, selective attention, visuo-spatial working memory, and planning.

In the alertness training, the patient drove a motorcycle at varying speed along a winding road. The aim was to carefully observe the stretch of the road in front of him/her and to press the keyboard as quickly as possible when an obstacle appeared on the road (i.e., a level crossing closes, a tree falls unexpectedly in the driver’s path), in order to brake promptly before it.

In the selective attention task, the patient was an explorer on a boat along a river surrounded by a forest. During the journey, several animals appeared, including hippos, giraffes, elephants. The patient was asked to press the keyboard only when he/she saw the hippos.

In the spatial working memory task, the patient watched colorful butterflies in a natural environment. The butterflies flew over a meadow or sandy area. From time to time, one butterfly landed, and another started its flight and so on. Depending on the level of difficulty, the patient was asked to remember the position of the last butterfly, the second-to-last butterfly, the third-to-last butterfly and so on.

In the planning training, the patient saw a map of the city with nine buildings (e.g., post office, café, insurance office, cultural center). On the right side of the map, a box appeared in which pending and completed errands were listed. The patient was asked to accomplish several tasks in each building, formulating an appropriate strategy to decide in which order running the errands. The task difficulty varied according to the number of errands to be completed and the time spent.

### 2.6. Procedure

#### 2.6.1. Transcranial Direct Current Stimulation (tDCS)

tDCS was applied using a battery driven Eldith (neuroConn GmbH) Programmable Direct Current Stimulator with a pair of surface-soaked sponge electrodes (5 × 7 cm). Anodal stimulation consisted of 20 min of 2 mA direct current with the anode placed over the right DLPFC (F4 of the extended International 10–20 system for EEG electrode placement) and the cathode (the reference electrode) above the contralateral frontopolar cortex (Fp1). For sham stimulation, the same electrode position was used. The current was ramped up to 2 mA and slowly decreased over 30 s to ensure the typical initial tingling sensation [73]. The order of conditions was randomized across subjects. Half of the participants started with the anodal condition and the remaining half with the sham condition. There were four weeks of intersession interval between the two experimental conditions. Thus, after four weeks, the order of condition was inverted. For each experimental condition (anodal vs. sham), the rehabilitative program consisted of 10 one-hour sessions over two weeks (Monday-Friday, weekends off, Monday-Friday). Although tDCS stimulation was delivered from the beginning of the cognitive training up to 20 min, the cognitive training lasted 1 h per day. At the end of each treatment condition (anodal vs. sham) and after four weeks (follow-up), the neuropsychological battery was readministered to all patients. During the training, none of the participants noticed differences in the intensity of sensation between the two stimulation conditions (anodal vs. sham), not being aware of what condition they were performing [74].

#### 2.6.2. Cognitive Treatments

The cognitive treatment was administered through the Cogniplus software (Schuhfried). During each one-hour session, all participants underwent four types of training: alertness, selective attention, visuo-spatial memory, and planning presented in randomized order.

#### 2.6.3. Data Analysis

Before, after the treatment and at follow-up (FU), the patients’ performance was evaluated by comparing the mean score obtained in the alertness, selective attention, visuo-spatial working memory, and planning training. Data were analyzed using STATISTICA10 software. The Shapiro–Wilk test was applied which revealed a normal distribution of the data. Four repeated measures ANOVAs were performed separately for the four types of training. For each analysis, two “within” factors were considered: CONDITION (anodal vs. sham) and TIME (baseline (T0) versus end of treatment (T10) versus follow up (FU)). The post-hoc Bonferroni test was conducted on the significant effects observed in the ANOVA. The values of *p* ≤ 0.05 were considered statistically significant. Before and after each treatment condition, the patients’ responses to the different re-administration of the standardized language tests (EDL and BADA), CADL-2 test, visual search test, Smirni subtest and TOL test were also analyzed using χ2-test.

## 3. Results

### 3.1. Accuracy Data

#### 3.1.1. Alertness

The analysis showed no significant effect of CONDITION (anodal versus Sham, F (1,19) = 1.51, *p* = 0.23, partial η^2^ = 0.07 and observed power = 0.21), but a significant effect of TIME [Baseline (T0) versus End of treatment (T10) versus Follow-up (F/U), F (2,38) = 360.40, *p* ≤ 0.001, partial η^2^ = 0.95, and observed power =1.000]. The interaction CONDITION × TIME was not significant (F (2,38) = 1.76, *p* = 0.19, partial η^2^ = 0.08 and observed power = 0.35).

#### 3.1.2. Selective Attention

**Figure 1:**
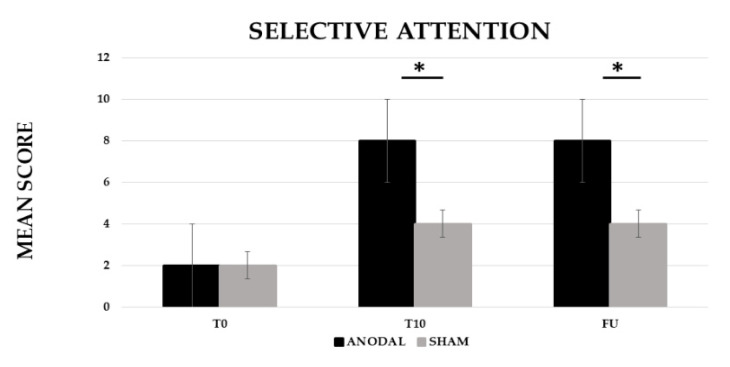
Mean score in the selective attention training at baseline (T0), at the end of treatment (T10) and at follow-up (F/U, 1 month after the end of treatment) for the anodal and sham condition, respectively. Sig. ANOVA: * *p* ≤ 0.001.

The analysis showed a significant effect of CONDITION (anodal versus Sham, F (1,19) = 222.13, *p* ≤ 0.001, partial η^2^ = 0.92 and observed power = 1.000) and of TIME (Baseline (T0) versus End of treatment (T10) versus Follow-up (F/U), F (2,38) = 427.84, *p* ≤ 0.001, partial η^2^ = 0.96 and observed power = 1.000). The interaction CONDITION × TIME was also significant (F (2,38) = 199.19, *p* ≤ 0.001, partial η^2^ = 0.91 and observed power = 1.000). The Bonferroni’s post-hoc test revealed that, while no significant differences emerged in the mean score between the two conditions at T0 (anodal 2 versus sham 2, *p* = 1), the mean score was significantly greater in the anodal than in the sham condition at T10 (anodal 8 versus sham 4, *p* ≤ 0.001) and persisted at F/U (anodal 8 versus sham 4, *p* ≤ 0.001). Significant differences also emerged between T0 and T10 for the sham condition (2, *p* ≤ 0.001) (see Figure 1).

#### 3.1.3. Visuo-Spatial Working Memory

**Figure 2:**
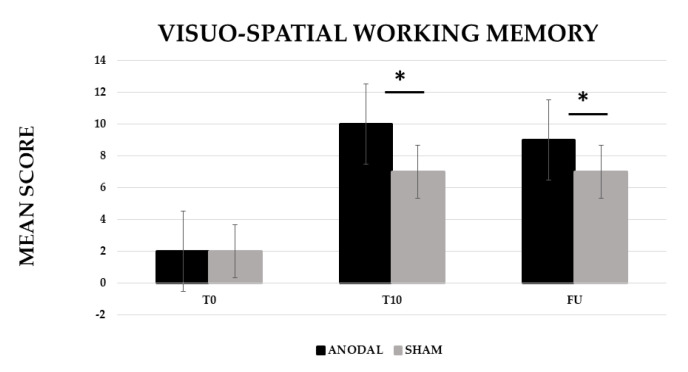
Mean score in the visuo-spatial working memory training at baseline (T0), at the end of treatment (T10) and at follow-up (F/U, 1 month after the end of treatment) for the anodal and sham condition, respectively. Sig. ANOVA: * *p* ≤ 0.001.

The analysis showed a significant effect of CONDITION (anodal versus Sham, F (1,19) = 20.29, *p* ≤ 0.001, partial η^2^ = 0.52 and observed power = 0.99) and TIME [Baseline (T0) versus End of treatment (T10) versus Follow-up (F/U), F (2,38) = 169.29, *p* ≤ 0.001, partial η^2^ = 0.90 and observed power = 1.000]. The interaction CONDITION × TIME was also significant (F (2,38) = 18.99, *p* ≤ 0.001, partial η^2^ = 0.50 and observed power = 0.99). The Bonferroni’s post-hoc test revealed that, while no significant differences emerged in the mean score between the two conditions at T0 (anodal 2 versus sham 2, *p* = 1), the mean score was significantly greater in the anodal than in the sham condition at T10 (anodal 10 versus sham 7, *p* ≤ 0.001) and persisted at F/U (anodal 9 versus sham 7, *p* ≤ 0.001). Significant differences also emerged between T0 and T10 for the sham condition (5, *p* ≤ 0.001) (see Figure 2).

#### 3.1.4. Planning

**Figure 3:**
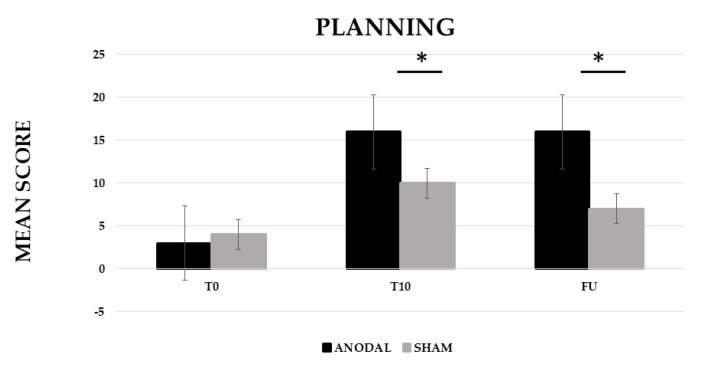
Mean score in the planning training at baseline (T0), at the end of treatment (T10) and at follow-up (F/U, 1 month after the end of treatment) for the anodal and sham condition, respectively. Sig. ANOVA: * *p* ≤ 0.001.

The analysis showed a significant effect of CONDITION (anodal versus Sham, F (1,19) = 201.06, *p* ≤ 0.001, partial η^2^ = 0.91 and observed power = 1.000) and TIME (Baseline (T0) versus End of treatment (T10) versus Follow-up (F/U), F (2,38) = 438.24, *p* ≤ 0.001, partial η^2^ = 0.96 and observed power = 1.000). The interaction CONDITION × TIME was also significant (F (2,38) = 237.05, *p* ≤ 0.001, partial η^2^ = 0.93 and observed power = 1.000). The Bonferroni’s post-hoc test revealed that, while no significant differences emerged in the mean score between the two conditions at T0 (anodal 3 versus sham 4, *p* = 1), the mean score was significantly higher in the anodal than in the sham condition at T10 (anodal 16 versus sham 10, *p* ≤ 0.001) and persisted at F/U (anodal 16 versus sham 9, *p* ≤ 0.001). Significant differences also emerged between T0 and T10 for the sham condition (6, *p* ≤ 0.001) (see Figure 3).

Interestingly, the χ^2^-test also revealed that, when the training was combined with anodal stimulation, all patients significantly improved not only in the CADL-2 test but also in oral noun and verb naming and in oral and written comprehension of sentences (see Table 3).

Moreover, after anodal stimulation, eleven out of twenty patients further improved their performance in the visual search test, while nine and fifteen patients passed the cut-off scores in the Smirni and TOL tests, respectively. These changes persisted at F/U. No changes were observed in the Corsi test whose score was already above the cut-off in all patients (see Table 4).

## 4. Discussion

In the present study, we investigated whether different types of executive function training combined with tDCS would enhance functional communication in twenty persons with chronic severe aphasia. After the training, an improvement in selective attention, spatial working memory and planning abilities was found in both stimulation conditions (anodal vs. sham) but it was greater in the anodal condition compared to sham. More importantly, this improvement persisted one month after the intervention. Thus, the executive training alone exerted its own effectiveness, but the recovery process was further improved after anodal tDCS. No differences were found between the two conditions in the alertness task. This last result argues against an explanation simply due to enhanced cognitive arousal which should have also influenced the alertness task. Interestingly, after anodal stimulation, a significant improvement was also observed in functional communication, in noun and verb naming, in auditory and written language comprehension tasks and in different executive functions tests.

As stated in the Introduction, in persons with chronic severe aphasia, language skills might result dramatically impaired even years after the onset of the disease. This situation often impacts also on the person’s ability to rely on functional communication. That is, the ability to effectively communicate his/her own needs in social contexts making use of compensatory strategies which allow to bypass the person’s verbal limitations [75,76,77]. Indeed, all individuals can express their communicative intentions not only through language, but also through extralinguistic means such as hand gestures, body movements or facial expressions which are intentionally expressed to convey a message. The impact of inadequate strategic competence prevents severe aphasic people to maintain successful social relations and to pursue life goals [78]. Consequently, in addition to the assessment of formal aspects of language (phonological, lexical, and grammatical domains), in persons with severe aphasia, the adjunct of a functional communication scale is particularly relevant to test their communicative abilities.

In the present study, together with standardized language tests, we administered the Communication Activities of Daily Living scale (CADL 2) [68] which, among the different communication abilities assessment tools, is considered a valid ecological battery for functional communication assessment [79]. The CADL 2 assesses a person’s communication abilities in activities of daily life by asking him/her to simulate communication acts in hypothetical natural environments (e.g., going to the doctor, making a phone call, asking for directions). It has been widely employed in the assessment of everyday language abilities in persons with aphasia [80,81] and in the evaluation of intensive language rehabilitation programs [82]. Interestingly, our results clearly showed that, while, before the training, all patients obtained very low percentage scores in the CADL-2, after two weeks of treatment, all of them reached an average percentage score but only when the training was combined with anodal tDCS. Thus, although the training was effective, the greater improvement obtained in the anodic condition was reflected in a positive change in the CADL-2 only in this condition. Similar results, although not for all patients, were obtained in the tests of attention, spatial memory, and planning skills.

As already mentioned, in recent years, there has been increasing interest in understanding the role of executive processes (e.g., cognitive control, attention, working memory) in the recovery of post-stroke language deficits [62,83,84]. The cerebral regions involved in executive function processing have been shown to be recruited in language tasks in post-stroke aphasia and in healthy subjects [85]. Researchers have also found that executive regions play a major role in recovery from aphasia [86]. Indeed, to enact goal-directed behavior and to respond to novel and challenging tasks of everyday life, people should make use of different cognitive components, such as cognitive flexibility, working memory and attention which rely on executive functions processing. Executive functions have been linked to pragmatic abilities and to social behavior [87] as they are involved in planning, monitoring, and inhibiting the discourse and in social exchanges. Moreover, intact executive functions system seems to be crucial to adaptive, motivated, and effective communication [88,89]. Impaired executive functions processing has been described in aphasic stroke patients and it has been shown to have a negative impact on rehabilitation outcomes [15,16,20,90,91,92], functional communication [25], and quality of life [93]. However, in most of these studies, the relationship between executive control and functional communication has been investigated only by assessing the aphasic performance through neuropsychological tests, including measures of functional communication, executive functional ability, and language impairment [25,26,94,95]. Based on these findings, it appears that higher levels of executive functioning are linked to better functional communication [21] and conversational skills [96] and greater cognitive flexibility has been significantly correlated to better strategy use in functional communication tasks [97]. Indeed, in the Olsson and colleagues [25,26] studies, most of the aphasic patients (79%) presented with executive functions deficits with the nonverbal participants more severely affected than the verbal group. Few recent studies have directly investigated if spared executive control is an important predictor of treatment gains. In the Simic et al. study [35], ten patients with mild to severe aphasia were treated three times a week for five weeks with a phonological naming therapy. Difference scores in naming accuracy of treated and untreated words served as the primary outcome measures. Results showed that individuals with better executive functions abilities showed better maintenance of treated words at four and eight- weeks follow-ups.

As far as we know, to date, only one study has emphasized the importance of strengthening executive control through prefrontal tDCS to facilitate lexical retrieval and verbal fluency in aphasia [62]. However, until now, no studies have investigated the impact of executive function training combined with tDCS on functional communication.

In our study, after the training, all patients improved in their functional communication skills and in several language and executive functions tasks, indicating an improvement in executive control enhanced functional communication. Interestingly, as also noted in previous works (see [37] for a review), these generalization effects on untreated tasks were present only after anodal stimulation. Indeed, the sham condition impacted only on the cognitive treatments as such. These results might be ascribed to the severity and chronicity of the functional communication profile observed, at baseline, in all patients. Indeed, since the treatment lasted only ten days, the hypothesis might be advanced that the executive function training alone was insufficient to improve functional communication. On the contrary, interestingly, the same number of training sessions combined with anodal tDCS over the same time period exerted beneficial effects on the treated functions and generalized to functional communication and to the language domain. Thus, similar to previous results (see [37] for a review), combining tDCS with cognitive training boosted cognitive recovery overcoming the difficulties caused by the severity of the deficit. These findings are, thus, very promising, as ten days of tDCS produced generalization effects over untreated functions (i.e., functional communication and language) which lasted up to one month which were not obtained in the absence of stimulation.

It is well known that executive functions are dependent on the prefrontal lobes which are strongly interconnected with other cortical areas and subcortical structures ([98,99] among which the frontal areas. In our study, we choose to stimulate the right DLPFC due to its role in selective attention, spatial working memory and planning abilities. Indeed, most of the recent tDCS literature have found that anodal tDCS over the right DLPFC enhances these skills [53,54,55,56,57]. Moreover, since all of our patients had extensive damage to the left language areas, we reasoned that stimulation of the right DLPFC would also have positive effects on language tasks. Indeed, even if the exact underlying tDCS mechanisms in our study remain largely speculative, the hypothesis might be advanced that, due to the strong interconnections between the prefrontal and the frontal regions [85,86,98,99], anodal tDCS over the right DLPFC has enhanced activation on the right frontal cortex which, in turn, serves as a supportive area for the observed recovery. Indeed, to date, several neuroimaging studies have already shown functional connectivity changes on cortical activity within the lesioned left hemisphere [100,101,102] and in the contralateral right homologues [103,104,105] due to tDCS treatment.

## 5. Conclusions

We are aware that our study has some limitations. The major ones are the small sample size and the lack of longitudinal follow-ups and of neuroimaging recordings. However, considering these limitations, we believe that our study highlights several important aspects to be considered when making treatment decisions for people with severe aphasia. First of all, it points to the possibility of training cognitive functions other than language. Indeed, from a connectionist perspective which considers the language system as part of a network largely distributed across the brain, this allows clinicians to plan different cognitive treatments which, in turn, facilitate aphasia recovery. It also emphasizes the need to assess functional communication skills, the recovery of which, even in the most severely affected patients, will allow the patient to socially interact in everyday life contexts. Finally, it confirms several previous reports which suggest that post-stroke aphasics in the chronic phase can still benefit from combining the treatment with tDCS.

In conclusion, although future studies are needed to deepen our understanding of the role of executive control on functional communication and the underlying neural mechanisms by which tDCS affects verbal performance, we believe that our results are promising since, for the first time, they suggest that executive function training can positively impact functional communication in severe chronic aphasia.

## Figures and Tables

**Table 1:** Sociodemographic and clinical data of the twenty non-fluent aphasic patients (Esame del Linguaggio II, [65], cut-off 100%; Token test (TT), [66], cut-off score 29/36). In each test, the percentages of correct responses are reported except for the Token Test whose score cannot be converted in percentage.

P	Sex	Age	Ed. Level	Time Post- Onset	Stroke Type	Lesion Side LH	Oral NN	Oral VN	Writ NN	Writ VN	WR	NWR	W Read	NW Read	WD	NW D	TT
1	M	50	8	4 y	I	FTP	5	5	0	0	20	15	12.5	5	0	0	5
2	M	58	13	3 y	I	FTP	0	5	0	0	10	0	10	0	0	0	5
3	M	60	8	1 y	I	FT	0	0	0	0	17.5	10	10	5	0	0	4
4	F	72	8	1 y	I	FTI	0	0	0	0	0	0	10	0	0	0	5
5	M	66	13	1 y	I	FT	0	0	0	0	20	12.5	15	5	0	0	4
6	M	67	17	2 y	I	FT	0	0	0	0	0	0	0	0	0	0	5
7	F	58	13	2 y	H	FTI	5	5	0	0	20	10	10	0	0	0	5
8	F	72	8	1 y	I	FTP	0	0	0	0	0	0	0	0	0	0	7
9	M	59	13	2 y	I	FTI	0	5	0	0	17.5	10	10	0	0	0	5
10	M	59	17	6 mo	I	FTI	5	0	0	0	0	0	0	0	0	0	4
11	F	72	8	2 y	I	FTP	0	0	0	0	15	5	12.5	5	0	0	7.5
12	M	54	17	6 mo	I	FTP	5	0	0	0	15	5	15	0	0	0	4
13	F	58	13	3 y	I	FTP	5	0	0	0	15	5	10	0	0	0	5
14	F	65	17	1 y	I	FT	5	0	0	0	10	0	10	0	0	0	4
15	M	69	8	2 y	I	FTP	5	5	0	0	0	0	0	0	0	0	4
16	M	55	13	3 y	I	FT	0	0	0	0	0	0	0	0	0	0	6
17	F	52	17	6 mo	I	FTP	0	5	0	0	0	0	0	0	0	0	5
18	F	61	8	1 y	I	FTI	5	0	0	0	0	0	0	0	0	0	5
19	F	53	13	2 y	I	FTP	0	0	0	0	0	0	0	0	0	0	4
20	F	68	13	4 y	I	FTP	0	5	0	0	0	0	0	0	0	0	5

**Legend:** P = Participants; M = male; F = female; Ed. Level = Educational Level; y = year/years; mo = months; I = Ischaemic; H = Haemorrhagic; LH = Left hemisphere; FTP = fronto-temporo-parietal; FT = fronto-temporal; FTI = fronto-temporo-insular; Oral NN = Noun Naming; Oral VN = Verb Naming; Writ NN = Written Noun Naming; Writt VN = Written Verb Naming; WR = Word Repetition; NWR = Nonword Repetition; W Read = Word Reading; NW Read = Nonword Reading; WD = Word under Dictation; NWD = Nonword under Dictation; TT = Token Test.

**Table 2:** Clinical data of the twenty non-fluent aphasic patients in Auditory and Written sentence comprehension of the BADA test (Batteria per l’Analisi dei Deficit Afasici, [67]), in the CADL-2 test (Communication Activities of the Daily Living [68]; 0–23 low percentage scores, 24–77 average percentage score, 78–100 high percentage score), in the Visual Search ([69] (cut-off score 30), in the Corsi Span Backward ([70] (cut-off score 3,08), in the Smirni test ([71] (cut-off percentage score > 10) and in the TOL test (Tower of London, [72] (cut-off percentage score 80). The percentages of correct responses are reported for all tests except for Auditory and Written sentence comprehension of the BADA test, the Corsi test and the Visual Search whose score cannot be converted in percentage.

P	Auditory Sentence Compreh	Written Sentence Compreh	CADL-2	Visual Search	Corsi Backward	Smirni	TOL
1	5	5	20	31	4	5	75
2	4	2	23	31	5	10	72
3	4	3	21	32	5	10	78
4	3	2	16	34	4	5	70
5	2	4	17	33	4	5	78
6	3	4	15	32	4	5	72
7	5	5	23	32	4	10	71
8	2	2	20	34	4	5	76
9	4	4	19	33	5	10	76
10	3	3	22	32	4	10	73
11	4	2	18	31	4	5	70
12	5	4	19	31	4	10	71
13	5	3	20	34	5	10	70
14	4	2	17	32	5	5	71
15	3	4	18	31	4	5	76
16	2	4	21	34	4	5	74
17	3	5	19	31	4	5	72
18	3	5	20	33	4	5	73
19	5	3	21	34	4	10	70
20	4	4	22	31	5	10	78

**Legend:** P = Participants; Compreh = Comprehension; CADL-2 = Communication Activities of the Daily Living; TOL = Tower of London.

**Table 3:** Correct Responses in the Different Language Tasks (Esame del Linguaggio II(EDL), [65]; Battery for the Analysis of Aphasic Disorders test (BADA, [67]) and in the Communication Activities of the Daily Living test (CADL-2, [68]), at Baseline (T0), at the End of Treatment (T10), and at Follow up (FU) for the real and sham condition, respectively.

P	C	ORAL NN			ORAL VN			AUDITORY SENT COMP			WRITTEN SENT COMP			CADL-2		
		T0	T10	FU	T0	T10	FU	T0	T10	FU	T0	T10	FU	T0	T10	FU
**REAL FIRST**																
1	R	5	30 ^	30	5	30 ^	30	5	17 **	17	5	16 *	14	20	48 ^	42
	S	30	32.5	25	30	35	30	17	19	16	16	18	14	48	50	40
3	R	0	17.5 ^	15	0	12.5 ^	15	4	15 *	13	3	13 *	13	21	50 ^	50
	S	17.5	17.5	15	12.5	15	12.5	15	17	14	13	16	14	50	54	52
5	R	0	15 ^	15	0	10 **	10	2	14 *	13	4	13 *	11	17	36 **	38
	S	15	15	10	10	10	10	14	14	12	13	14	13	36	40	38
7	R	5	20 **	20	5	40 ^	42.5	5	15 *	15	5	12	11	23	55 ^	50
	S	20	25	20	40	45	40	15	16	15	12	11	9	55	59	48
9	R	0	12.5 ^	15	5	30 ^	25	4	20 ^	20	4	18 **	18	19	36 **	40
	S	12.5	15	15	30	30	20	20	19	19	18	19	18	36	40	40
11	R	0	10 **	10	0	20 ^	20	4	12	13	2	11 *	12	18	40^	40
	S	10	15	15	20	25	25	12	13	13	11	11	11	40	46	44
13	R	5	15 *	15	0	10 **	10	5	13	11	3	14 **	15	20	51 ^	51
	S	15	20	17.5	10	10	7.5	13	13	9	14	16	16	51	55	50
15	R	5	20 **	20	5	30 ^	32.5	3	15 **	15	4	16 **	16	18	39 **	36
	S	20	22.5	20	30	30	30	15	18	18	16	19	16	39	43	40
17	R	0	20^	17.5	5	25 ^	25	3	10	11	5	13	12	19	44 ^	48
	S	20	30	25	25	30	30	10	13	13	13	13	11	44	46	46
19	R	0	17.5 ^	15	0	20 ^	17.5	5	14 *	14	3	13 *	14	21	48 ^	48
	S	17.5	25	20	20	20	15	14	13	13	13	15	14	48	47	47
**SHAM FIRST**																
2	S	0	0	0	5	10	10	4	7	7	2	4	5	23	27	23
	R	0	15 ^	12.5	10	35 ^	30	7	18 *	15	4	14 *	14	27	46 **	40
4	S	0	5	5	0	0	0	3	8	8	2	7	6	16	20	20
	R	5	25 ^	22.5	0	20 ^	15	8	18 *	18	7	19 *	17	20	44 ^	44
6	S	0	0	0	0	0	0	3	7	6	4	5	5	15	18	20
	R	0	10 **	10	0	0	0	7	17 *	14	5	14 *	13	18	42 ^	44
8	S	0	0	0	0	0	0	2	4	4	2	4	4	20	25	25
	R	0	12.5	15	0	15 ^	15	4	15 *	15	4	19 ^	18	25	53 ^	50
10	S	5	10	10	0	5	5	3	8	8	3	5	4	22	25	22
	R	10	30 ^	30	5	25 ^	22.5	8	23 **	21	5	14 *	13	25	55 ^	50
12	S	5	7.5	5	0	0	0	5	10	10	4	7	7	19	23	23
	R	7.5	20 *	20	0	20 ^	20	10	20	21	7	18 *	19	23	47 ^	47
14	S	5	10	10	0	0	0	4	6	5	2	4	4	17	22	20
	R	10	25 **	20	0	20 ^	17.5	6	16 *	15	4	15 **	16	22	46 ^	42
16	S	0	5	5	0	10 **	10	2	4	4	4	6	5	21	26	24
	R	5	22.5 ^	20	10	35 ^	30	4	14 *	14	6	16 *	14	26	53 ^	48
18	S	5	10	10	0	0	0	3	5	6	5	9	9	20	22	20
	R	10	25 **	25	0	15 ^	15	5	17 **	19	9	18	19	22	41 **	41
20	S	0	0	0	5	10	10	4	7	7	4	8	7	22	24	24
	R	0	10 **	7.5	10	30 ^	25	7	19 *	19	8	20 *	19	24	53 ^	53

**Legend:** P = Participants; C = Condition; Oral NN = Noun Naming; Oral VN = Verb Naming; Auditory Sent Comp = Auditory Sentence comprehension; Written Sent Comp = Written Sentence Comprehension; CADL-2 = Communication Activities of the Daily Living; R = Real stimulation; S = Sham stimulation; * = *p* < 0.05; ** = *p* < 0.01; ^ = *p* < 0.001.

**Table 4:** Correct Responses in the Different Cognitive Tasks (Visual Search, [69]; Smirni test, [71]; Tower of London (TOL) [72] at Baseline (T0), at the End of Treatment (T10) and at Follow up (FU) for the real and sham condition, respectively.

P	C	VISUAL SEARCH			SMIRNI			TOL		
		T0	T10	FU	T0	T10	FU	T0	T10	FU
**REAL FIRST**										
1	R	31	40	40	5	5	5	75	90 **	85
	S	40	40	41	5	5	5	90	90	80
3	R	32	41	39	10	50 ^	50	78	92 **	90
	S	41	40	38	50	50	50	92	92	90
5	R	33	34	33	5	5	5	78	91 *	94
	S	34	34	33	5	5	5	94	94	92
7	R	32	33	34	10	25 *	25	71	79	75
	S	33	35	34	25	25	25	79	73	73
9	R	33	45 *	46	10	25 *	25	76	79	76
	S	45	46	48	25	25	25	79	76	76
11	R	31	40	41	5	5	5	70	88 **	89
	S	40	39	40	5	5	5	88	88	90
13	R	34	43	45	10	50 ^	50	70	92 ^	95
	S	43	45	44	50	50	50	92	95	95
15	R	31	42	41	5	5	5	76	88 *	85
	S	42	41	40	5	5	5	88	86	83
17	R	31	32	32	5	5	5	72	74	72
	S	32	32	31	5	5	5	74	73	72
19	R	34	35	34	10	25 *	25	70	85 *	84
	S	35	34	33	25	25	25	85	88	84
**SHAM FIRST**										
2	S	31	31	32	10	10	10	72	72	80
	R	31	31	31	10	25 *	25	72	88 *	89
4	S	34	35	38	5	5	5	70	75	73
	R	35	44	33	5	5	5	75	88 *	89
6	S	32	33	33	5	5	5	72	72	71
	R	33	41	43	5	5	5	72	69	70
8	S	34	34	33	5	5	5	76	76	80
	R	34	34	34	5	5	5	76	89 *	91
10	S	32	33	34	10	10	10	73	78	85
	R	33	34	35	10	50 ^	50	78	100 ^	95
12	S	31	32	31	10	10	10	71	4	75
	R	32	32	32	10	50 ^	50	74	85 *	80
14	S	32	34	37	5	5	10	71	75	80
	R	34	44	45	5	10	10	75	87 **	87
16	S	34	38	35	5	5	5	74	76	75
	R	38	40	38	5	5	5	76	75	75
18	S	33	36	40	5	5	10	73	71	77
	R	36	47	48	5	10	10	71	86 *	88
20	S	31	34	38	10	10	10	78	78	83
	R	34	45	46	10	25 *	25	78	93 **	95

**Legend**: P = Participants; C = Condition; TOL = Tower of London; R = Real stimulation; S = Sham stimulation; * = *p* < 0.05; ** = *p* < 0.01; ^ = *p* < 0.001.

## Data Availability

The data presented in this study are available on request from the corresponding author. The data are not publicly available due to ethical and privacy restrictions.

## Supplementary Materials

The following supporting information can be downloaded at: https://www.mdpi.com/article/10.3390/brainsci12091265/s1, Figure S1: CONSORT Flow Diagram.
