# Supplementary figures and images for: Does Executive Function Training Impact on Communication? A Randomized Controlled tDCS Study on Post-Stroke Aphasia
Source: Brain Sci. 2022 Sep 19;12(9):1265. doi: 10.3390/brainsci12091265 (PMC9497246; doi:10.3390/brainsci12091265)

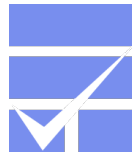

# CONSORT

TRANSPARENT REPORTING of TRIALS

## CONSORT Flow Diagram

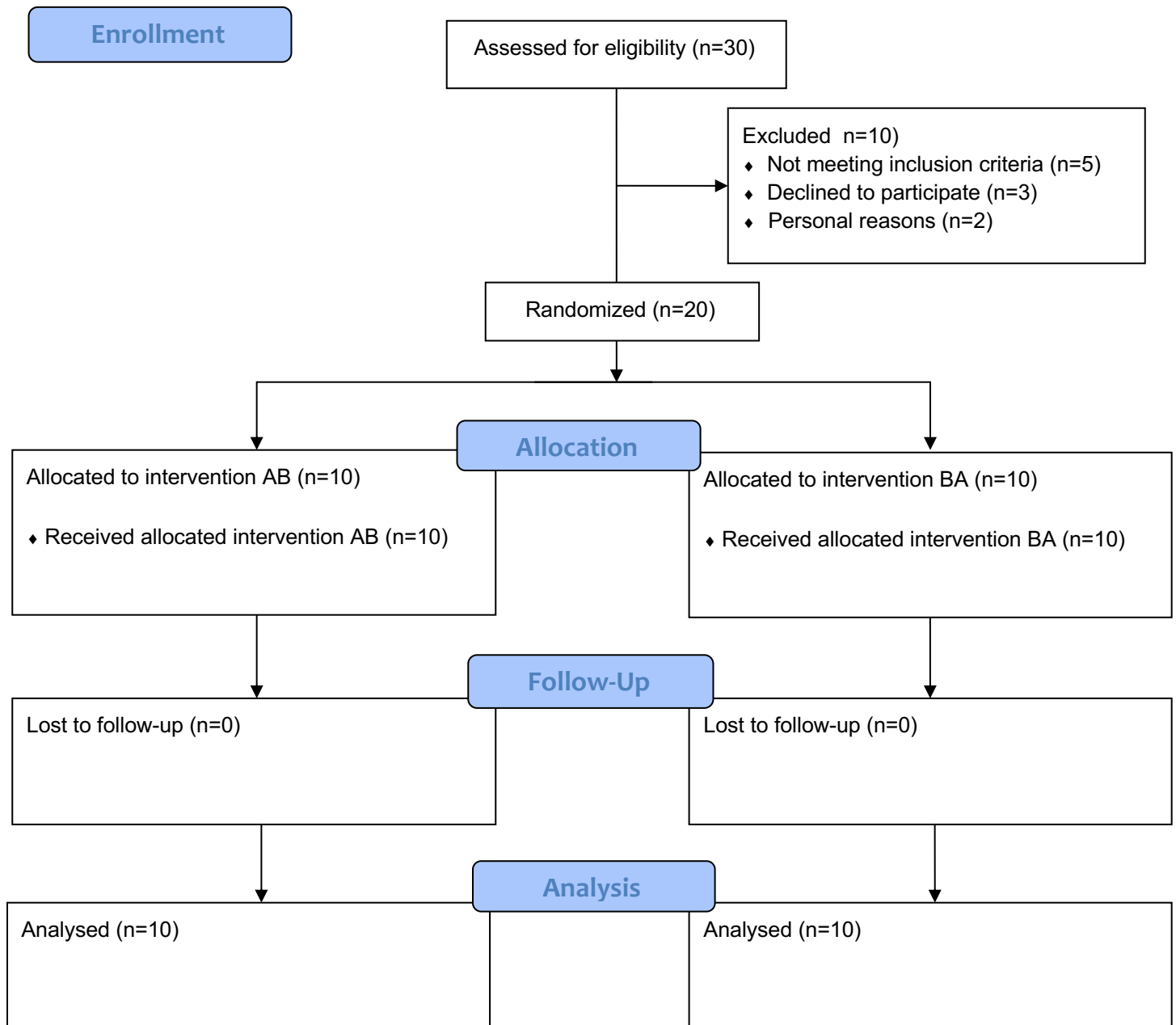

Supplement: Supplementary file 1 [file brainsci-12-01265-s001.zip › brainsci-1914967-supplementary.pdf]
